# A potential therapeutic effect of catalpol in Duchenne muscular dystrophy revealed by binding with TAK1

**DOI:** 10.1002/jcsm.12581

**Published:** 2020-08-31

**Authors:** Dengqiu Xu, Lei Zhao, Jingwei Jiang, Sijia Li, Zeren Sun, Xiaofei Huang, Chunjie Li, Tao Wang, Lixin Sun, Xihua Li, Zhenzhou Jiang, Luyong Zhang

**Affiliations:** ^1^ Jiangsu Key Laboratory of Drug Screening China Pharmaceutical University Nanjing China; ^2^ Department of Neurology Children's Hospital of Fudan University Shanghai China; ^3^ Center for Drug Research and Development Guangdong Pharmaceutical University Guangzhou China; ^4^ Jiangsu Center for Pharmacodynamics Research and Evaluation China Pharmaceutical University Nanjing China; ^5^ Key Laboratory of Drug Quality Control and Pharmacovigilance China Pharmaceutical University Nanjing China

**Keywords:** TAK1, Duchenne muscular dystrophy, Catalpol, Fibrosis, Myogenesis

## Abstract

**Background:**

Duchenne muscular dystrophy (DMD) is a progressive muscle disease caused by the loss of dystrophin, which results in inflammation, fibrosis, and the inhibition of myoblast differentiation in skeletal muscle. Catalpol, an iridoid glycoside, improves skeletal muscle function by enhancing myogenesis; it has potential to treat DMD. We demonstrate the positive effects of catalpol in dystrophic skeletal muscle.

**Methods:**

*mdx* (loss of dystrophin) mice (*n* = 18 per group) were treated with catalpol (200 mg/kg) for six consecutive weeks. Serum analysis, skeletal muscle performance and histology, muscle contractile function, and gene and protein expression were performed. Molecular docking and ligand–target interactions, RNA interference, immunofluorescence, and plasmids transfection were utilized to explore the protective mechanism in DMD by which catalpol binding with transforming growth factor‐β–activated kinase 1 (TAK1) in skeletal muscle.

**Results:**

Six weeks of catalpol treatment improved whole‐body muscle health in *mdx* mice, which was characterized by reduced plasma creatine kinase (*n* = 18, −35.1%, *P <* 0.05) and lactic dehydrogenase (*n* = 18, −10.3%, *P <* 0.05) activity. These effects were accompanied by enhanced grip strength (*n* = 18, +25.4%, *P <* 0.05) and reduced fibrosis (*n* = 18, −29.0% for hydroxyproline content, *P <* 0.05). Moreover, catalpol treatment protected against muscle fatigue and promoted muscle recovery in the tibialis anterior (TA) and diaphragm (DIA) muscles (*n* = 6, +69.8%, *P <* 0.05 and + 74.8%, *P <* 0.001, respectively), which was accompanied by enhanced differentiation in primary myoblasts from DMD patients (*n* = 6, male, mean age: 4.7 ± 1.9 years) and *mdx* mice. In addition, catalpol eliminated p‐TAK1 overexpression in *mdx* mice (*n* = 12, −21.3%, *P <* 0.05) and primary myoblasts. The catalpol‐induced reduction in fibrosis and increased myoblast differentiation resulted from the inhibition of TAK1 phosphorylation, leading to reduced myoblast trans‐differentiation into myofibroblasts. Catalpol inhibited the phosphorylation of TAK1 by binding to TAK1, possibly at Asp‐206, Thr‐208, Asn‐211, Glu‐297, Lys‐294, and Tyr‐293.

**Conclusions:**

Our findings show that catalpol and TAK1 inhibitors substantially improve whole‐body muscle health and the function of dystrophic skeletal muscles and may provide a novel therapy for DMD.

## Introduction

Duchenne muscular dystrophy (DMD) is the most common X‐linked progressive muscle disease in humans, affecting up to 1 in 3600 live male births, with about one third of cases attributable to new spontaneous mutations in the dystrophin gene.^1^ The early stages of DMD are characterized by the gradual degeneration and regeneration of muscle fibres, which are followed by a reduction in their regenerative ability, fibrosis, and the disruption of muscle tissue architecture.^2^ Clinically, patients usually develop signs from the age of 3–5 years, with progressive leg muscle weakness affecting walking; they die of respiratory or cardiac failure between the ages of 20 and 30 years.^3^ RNA‐modifying therapies only for some DMD patients, and no effective drug treatment for the disease is available. Dystrophin is an important cytoskeletal protein that helps the cytoskeleton of each muscle fibre connect to the underlying basal lamina. The alteration or loss of dystrophin forces excess calcium into the cell membrane, resulting in excess water content in the mitochondria; consequently, the affected skeletal muscle undergoes atrophy, mitochondrial dysfunction, and necrosis.^4^

Transforming growth factor‐β–activated kinase 1 (TAK1), a member of the MEK kinase (MAP3K) family, plays a key role in non‐canonical transforming growth factor (TGF)‐β receptor signalling. TAK1 is also a downstream signalling molecule and central mediator in many other pathways. Cytokines such as tumour necrosis factor (TNF)‐α and IL‐1 activate signalling cascades in inflammatory cells that involve MAP kinase signalling and lead to the activation of NF‐κB and c‐Jun‐N‐terminal kinase.^5^ Several members of the MAP 3K family play essential roles in the growth and atrophy of skeletal muscle.^6^, ^7^ TAK1 is thought to be a dominant profibrotic mediator in patients with DMD and *mdx* mice (a model of DMD). TAK1 is an important regulator of skeletal muscle fibre formation and plays important roles in cell division, proliferation, inflammation, and fibrosis.^8^, ^9^ The activation of TAK1 promotes fibrosis,^10^, ^11^ possibly because TAK1 activates the NF‐κB pathway of muscular dystrophy fibroblasts.^9^, ^11^, ^12^ TAK1 activation has been shown to promote the transformation of myoblasts into myofibroblasts.^13^ In young mice *in vivo*, TAK1 is expressed primarily in developing skeletal muscle; in adult mice, TAK1 expression is increased during skeletal muscle regeneration. The deletion of TAK1 in muscle blocks the regeneration of skeletal muscle satellite cells, indicating that TAK1 plays an important role in maintaining the regeneration of these cells.^14^ The differentiation of skeletal muscle cells is regulated by the MyoD family (e.g. MyoD and myogenin), and myogenin is essential for the differentiation of muscle cells. TAK1 specifically regulates the expression of the MyoD family, thereby affecting muscle cell differentiation.^15^ The expression of TAK1 is decreased during myoblast differentiation, and TAK1 activation inhibits this differentiation, suggesting that TAK1 is also involved in the regulation of myoblast differentiation.

Catalpol, an iridoid glycoside, is found mainly in the traditional Chinese medicinal herb *Rehmannia*. Catalpol has hypoglycemic, anti‐inflammatory, and antioxidant effects.^16^, ^17^ Previously, we showed that catalpol improves skeletal muscle function by activating MyoD/MyoG‐mediated myogenesis, which promotes the utilization of glucose in external tissues and has hypoglycemic effects.^18^ However, whether catalpol also has potential for the treatment of DMD remains unknown. Thus, this study explored the protective effect of catalpol in DMD and examined the underlying mechanism. Simvastatin, which is used widely for the reduction of total cholesterol, has been reported to dramatically reduce inflammation, fibrosis, and oxidative stress in *mdx* mice. We choose simvastatin as a positive drug to compare the protective effect of catalpol in *mdx* mice.

## Materials and methods

### Patients and study design

Muscle biopsies obtained from control donors with orthopaedic surgery (*n* = 6) and DMD donors (*n* = 6) matched by aged (2–8 years). All muscle biopsies obtained from Children's Hospital of Fudan University, whose diagnosis was established based on loss of dystrophin by immunohistochemistry and Western blot in Children's Hospital of Fudan University. In addition, fresh muscle biopsies (50–100 mg) were used to isolated primary myoblasts as described subsequently. The protocol was approved by the Institutional Review Board at Children's Hospital of Fudan University (Shanghai, China), and all participants provided written informed consent prior to initiation of study procedures. This study is registered clinical trial at Children's Hospital of Fudan University (NCT number: 2019‐244).

### Animal studies

All experimental procedures performed on mice were conducted in accordance with the Guide for the Care and Use of Laboratory Animals published by the US National Institutes of Health (Publication No. 85‐23, revised 1996) and Use Committee of China Pharmaceutical University (Nanjing, China; 2019–11‐006), and the use of the laboratory animals was approved by the Laboratory Animal Management Committee Office (Fuzhou, China). Dystrophin‐deficient C57BL/10ScSnJNju‐Dmdem3Cd4/Gpt (*mdx*) mice and C57BL/10ScSn/J strain controls were purchased from Nanjing University Animal Model Research Center (Nanjing, China). All comparisons were made between age‐matched male mice. Experiment 1: 54 *mdx* mice (*n* = 54, male mice, 6–7 weeks old) were randomly allocated into the DMD group, 200 mg/kg catalpol‐treated group, 20 mg/kg simvastatin‐treated group (*n* = 18 per group), and their normoglycaemic littermates (*n* = 18, male mice, 6–7 weeks old) as control group. Catalpol and simvastatin were orally administered for six consecutive weeks, while the control group and DMD group were treated with saline. At the end of the drug treatment, all mice were sacrificed, and tibialis anterior (TA), extensor digitorum longus (EDL), gastrocnemius (GAS), soleus (SOL), and diaphragm (DIA) muscle sections were collected. Experiment 2: Nine *mdx* mice (*n* = 9, male mice, 3–4 weeks old) and their normoglycaemic littermates (*n* = 9, male mice, 3–4 weeks old) were used to study the effects of short hairpin RNA (shRNA)‐TAK1 on DMD. All mice were anaesthetized using isoflurane. Right TAs were injected with 1.1 × 10^12^ vg/TA in 40 μL of AAV2/9 expressing shRNA‐TAK1, and the same dose of AAV2/9 expressing shRNA‐control was injected in the left TA. After 4 weeks, mice were euthanized. The TA muscle sections were used for histological and Western blot analysis.

### *In vivo* muscle strength test and wire grip test

Forelimb grip strength test was used to assay the grip strength of forelimb using a calibrated grip strength tester (YLS‐13A, Yiyan Bio. Shandong, China). All tests were performed as described previously.^19^ The grip strength test was performed as the mean of at least three repetitions. A wire test is a typical method to assess muscle strength and the whole‐body force in mice. For the wire grip test was performed at 2 days before mice were sacrificed. All mice were allowed to grasp a 2 mm diameter metal wire, and the length of time was recorded until the mice fell. The test score was calculated as the mean of at least three repetitions.

### Skeletal muscle contractile function

TA strength (specific force) and fatigue resistance were determined using a length control system (BL‐420, Techman Scientific, Chengdu, China). After determining the optimum length as descried previously,^18^ the TA then underwent a fatigue protocol, in which it was stimulated at 120 Hz (200 ms duration) every 2 s for a total of 2 min. After 1 min, fatigue recovery was detected every 2 min up to total 10 min after fatigue.

### Blood biochemical analysis

Blood was collected from the inferior vena cava before the mice were euthanized. Blood samples were centrifuged at 3500 rpm for 15 min at 4°C and then used to measure serum creatine kinase (CK) and lactic dehydrogenase (LDH) activity by using an HITACHI7080 Automatic Clinical Analyzer (Tokyo, Japan).

### Isolated myoblasts

Human primary myoblasts from triceps muscle of DMD and mouse myoblasts from EDL or DIA muscle. All were twice washed with isotonic Dulbecco saline, minced with eye scissors to 1–2 mm^3^ fragments, and digested with 1.2 U/mL dispase type II (D4693, Sigma‐Aldrich, St. Louis, MO, USA) and 5 mg/ml collagenase type D (35799223; Roche Diagnostics Ltd., Shanghai, China) in DMEM incubated in a 95% oxygen and 5% CO_2_ incubator at 37°C for 1 h. Then, the threefold diluted tissue lysate was passed through a mesh for cell suspensions, precipitated, and washed with isotonic Dulbecco saline. All culture flasks were pre‐plated with 2% gelatin (Sigma‐Aldrich). The cells were seeded to a 25 cm^2^ culture flask in DMEM containing 20% foetal bovine serum (Gibco, Waltham, MA, USA), 1% chicken embryo extract, and 10% horse serum (Gibco) as described previously.^20^

### Single myofibre isolation

Mouse myofibres from EDL muscle were isolated by digesting muscles with 0.2% collagenase type I in DMEM incubated in a 95% oxygen and 5% CO_2_ incubator at 37°C for 45 min.^21^ To release myofibres, the bore glass pipette was used to flush the muscle with warm DMEM medium. Then, using the small pipette, transfer single myofibres in a new dish containing growth DMEM medium (20% foetal bovine serum, 1% chicken embryo extract, and 10% horse serum). Immunofluorescence staining was performed after myofibres treatment with 100 μM catalpol for 24 h.

### Real‐time quantitative polymerase chain reaction

Total RNA was isolated from the fresh TA muscle using TRIzol reagent and an RNeasy Kit (Vazyme Biotech, Nanjing, China). Complementary DNA was synthesized from 1 μg RNA in a final volume of 20 uL using complementary DNA Synthesis Kit (Vazyme Biotech, Nanjing, China) following the manufacturer's instructions. Real‐time polymerase chain reaction was performed using SYBR Green as previously reported.^22^ Gene expression was calculated by the ΔΔCT method using glyceraldehyde 3‐phosphate dehydrogenase (GAPDH) as the reference gene. All primer pairs used for the polymerase chain reaction are listed in *Table* S1.

### Histological analysis

Cryosections of TA, EDL, GAS, SOL, and DIA muscles were mounted on a slide, then transversely sectioned into 5 μm slices. The slides were stained with haematoxylin and eosin following standard protocols. For Masson's trichrome stain, DIA muscle was paraffin embedded and transversely sectioned into 8 μm‐thick slides, which were stained with Masson's trichrome. All slides were viewed, and photomicrographs were captured under light microscope (BX53, Olympus, Tokyo, Japan).

### Western blot analysis

Western blot analysis was performed as described previously.^20^ In brief, protein extracts were fractionated on 6–12% sodium dodecyl sulphate polyacrylamide gels and transferred onto polyvinylidene fluoride membranes (Millipore, Billerica, MA, USA) via electroblotting. The membranes were blocked in Tris‐buffered saline containing 5% bovine serum albumin for 90 min at room temperature. Next, membranes were incubated with the primary antibody at 4°C overnight. Then, membranes were incubated with the second antibody for 1 h, and signals were detected using a Bio‐Rad ECL system (Hercules, CA, USA). The bands were determined by densitometric analysis with Image‐Pro Plus software (Media Cybernetics, Rockville, MD, USA). The information of all antibodies was listed in *Table* S2.

### Immunofluorescence

Fresh TA muscle cryosections were mounted on a slide and blocked in blocking buffer for 60 min. Myoblasts, myofibres, and cryosections were fixed in PFA 4% for 1 h. Then, samples were incubated overnight with primary antibody against fibronectin (1:200; ab32419, Abcam); myosin heavy chain (MHC) (1:200; MF 20, Developmental Studies Hybridoma Bank, Iowa, USA); Pax7 (1:100; sc‐52903, Santa Cruz); fibroblast (1:100; sc‐73355, Santa Cruz); and myogenin (1:100; sc‐81648, Santa Cruz) at 4°C. Next, these samples were incubated with secondary antibody (Alexa Fluor 633, Alexa Fluor 488; Thermo, Waltham, MA, USA) for 1 h at room temperature. 4′,6‐Diamidino‐2‐phenylindole (DAPI) was used to visualize nuclei. The sections were viewed, and photomicrographs were captured under fluorescence microscope (FV1000, Olympus, Japan).

### Molecular docking and ligand–target interactions

Molecular docking is a widely accepted tool for lead identification; we used Autodock Vina to perform molecular docking. Catalpol and NG25 were docked to the detail crystal structure of the human TAK1 ligand‐binding domains (PDB code: O43318) under default parameters. Ligand–target interactions were calculated using Autodock Tools 1.5.6 under default parameters. Both the binding pockets and docking results were visualized using PyMOL as our previous report.^23^

### Short hairpin RNA

Validated plasmids encoding shRNA for mouse TAK1 (accession no. NM_172688) and negative control were purchased from Hanbio (Shanghai, China).^24^ TAK1 shRNA plasmid contained the targets sequence was TGAGAGGAAGGCTTTCATTGT. The sequence of control shRNA plasmid was GGAATCTCATTCGATGCATAC. All shRNA plasmids were amplified and purified by Hanbio (Shanghai, China).

### Plasmids and transfection assays

Human embryonic kidney (HEK) 293 T cells were used for the transfection assays. HEK293T cells grown in DMEM/F12 medium (Gibco, Grand Island, NY, USA) supplemented with 10% foetal bovine serum (Cat No: FSP500, ExCell Biotech, Shanghai, China) and 1% penicillin/streptomycin in a 5% carbon dioxide incubator at 37°C. HEK293T cells were exposed to TAK1 transfection for 24 h using 500 ng of a TAK1 DNA plasmid for each well of 12‐well plates. Catalpol (10, 100 μM) were added 24 h after transfection. After treatment 24 h with catalpol, cells were collected for western blot analysis.

### Statistical analysis

All obtained data were analysed with one‐way or two‐way analysis of variance followed by Tukey's multiple comparison post‐test using Graph Pad software (ver. 6.0; GraphPad Software Inc., La Jolla, CA, USA). Data are shown as mean ± standard deviation. The values with *P* < 0.05 were considered statistically significant.

## Results

### Catalpol protects dystrophic muscle from damage and fibrosis while improving muscle function

DMD is a progressive degenerative disease, and potential treatments need to be started at an early age. Thus, we first evaluated the effectiveness of catalpol treatment in terms of disease pathogenesis in *mdx* mice aged 4–6 weeks, the age of onset of muscle damage in these mice. We compared the efficacy of catalpol with that of simvastatin, a lipophilic statin with great potential to improve muscle function and health in DMD.^25^ Catalpol treatment partly restored reduced muscle function in *mdx* mice, as evidenced by a 29% increase in the maximal *in vivo* muscle grip strength (*P* < 0.05) and a 51% increase in the results of the wire test (*P* < 0.01). Whole‐body muscle health improved dramatically in catalpol‐treated and simvastatin‐treated *mdx* mice compared with *mdx* mice, as evidenced by a 36% reduction in the plasma CK level and a 10% reduction in the plasma LDH level (*Table* 1, *P* < 0.05). The *mdx* mice showed significant hypertrophy of all skeletal muscles examined (*Table* 1); catalpol partly reduced the hypertrophy of the TA and GAS muscles. The improved muscle function and health were validated by histological assessment of the TA muscle, which showed less inflammation in catalpol‐treated or simvastatin‐treated *mdx* mice (*Figure* S1). Furthermore, we found that the anti‐inflammatory effects of catalpol were lesser than those of simvastatin, indicated by CD68 immunofluorescence (*Figure* S1).

**TABLE 1:** Grip strength, duration of wire test, creatine kinase, and lactic dehydrogenase levels and TA, EDL, GAS, and SOL weights

	CON	DMD	Catalpol	Simvastatin
Grip strength (g)	83.5 ± 8.7	63.8 ± 10.9^***^	80.0 ± 10.2^#^	86.0 ± 8.7^##^
Duration of wire test (s)	154.7 ± 37.0	63.9 ± 13.5^***^	119.1 ± 25.6^##^	98.9 ± 22.0^#^
CK (U/L)	593 ± 237	2674 ± 384^***^	1738 ± 411^#^	1504 ± 238^#^
LDH (U/L)	484.7 ± 29.3	916.5 ± 60.6^***^	848.1 ± 48.3^#^	818.9 ± 48.7^#^
TA weight (mg)	103.4 ± 21.5	175.8 ± 46.5^***^	153.5 ± 16.8^##^	148.3.9 ± 15.7^##^
EDL weight (mg)	41.7 ± 6.1	58.0 ± 15.5	51.4 ± 14.5	40.0 ± 11.5
GAS weight (mg)	288.0 ± 33.4	386.7 ± 30.8^***^	339.1 ± 50.9^#^	355.2 ± 51.4
SOL weight (mg)	19.5 ± 2.3	23.8 ± 5.0	19.5 ± 1.9	20.9 ± 3.8

Values are presented as means ± standard deviations (*n* = 18 animals). CK, creatine kinase; LDH, lactic dehydrogenase; TA, tibialis anterior; EDL, extensor digitorum longus; GAS, gastrocnemius; SOL, soleus.

^***^ *P <* 0.001 vs. control (CON);

^#^ *P <* 0.05,

^##^ *P <* 0.01 *vs*. *mdx* mice (DMD).

**FIGURE 1:**
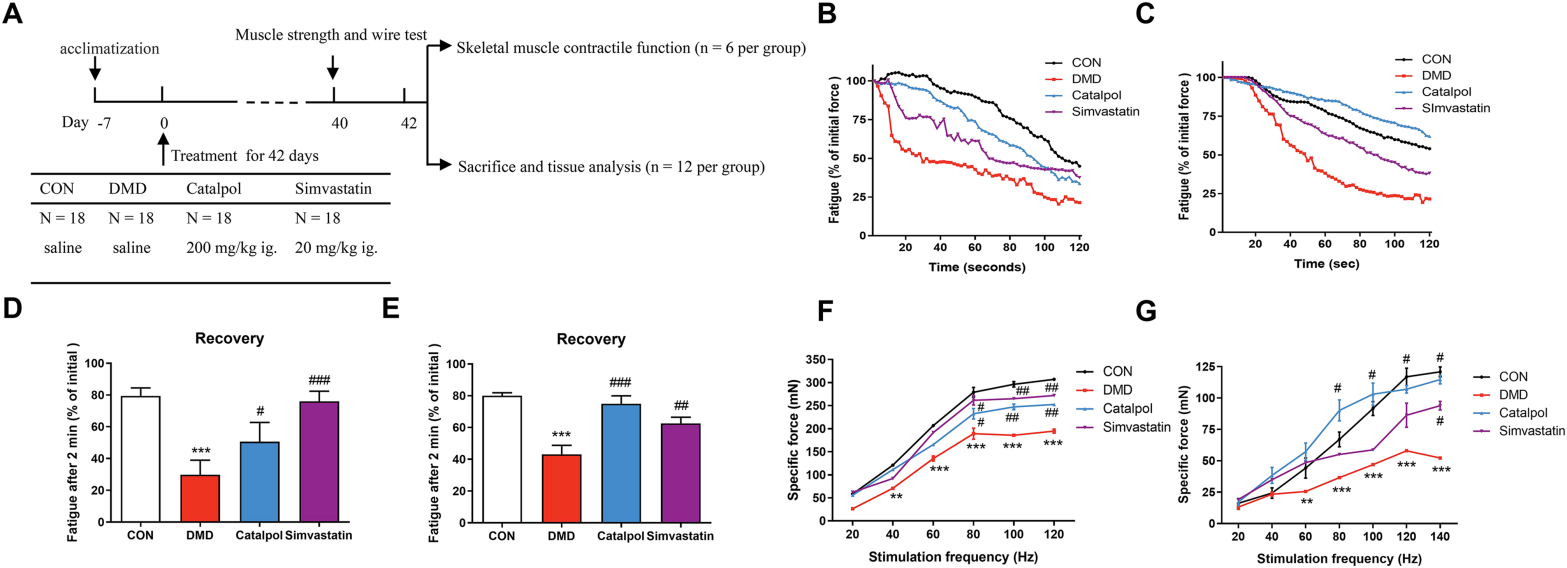
Catalpol protects against muscle fatigue and improves force recovery in *mdx* mice. (A) Schematic diagram of animal experiment design. Mice were treated with catalpol or simvastatin for 6 weeks from 4 weeks of age. (B, C) Representative traces of TA and DIA muscle force for all mice during 2 min of fatiguing contractions. (D, E) Pooled values of TA and DIA force after 1 min of fatigue. (F, G) Pooled values of TA and DIA force recovery after fatigue from 2 to 10 min. All data are shown as means ± standard deviation (*n* = 6 animals), ^***^ *P <* 0.001 vs. control (CON); ^#^ *P <* 0.05, ^##^ *P <* 0.01, ^###^ *P <* 0.001 vs. *mdx* mice (Duchenne muscular dystrophy).

In addition to the loss of grip strength, slowed force and increased muscle fatigue recovery are major causes of muscle weakness in DMD.^26^, ^27^ To examine the therapeutic effects of catalpol on skeletal muscle, fatigue in the TA and DIA muscles was measured. In *mdx* mice, force declined rapidly with fatigue in the TA and DIA muscles, by 76% and 79%, respectively, compared with the initial force (*P* < 0.05). However, the force declines in the catalpol and simvastatin groups were similar to that in the control group (*Figure* 1B and 1C). After 2 min of fatigue, *mdx* mice had significantly less force in the TA and DIA muscles than did control mice (*P* < 0.001). However, the catalpol‐treated and simvastatin‐treated *mdx* mice showed significant force recovery in the TA and DIA muscles (*P* < 0.01, *Figure* 1D and 1E). Moreover, the specific muscle force in the TA and DIA muscles was greater in catalpol‐treated and simvastatin‐treated *mdx* mice than in *mdx* mice, at 80–120 Hz stimulation frequencies (*P* < 0.01, *Figure* 1F and 1G), indicating an improvement in muscle performance.

**FIGURE 2:**
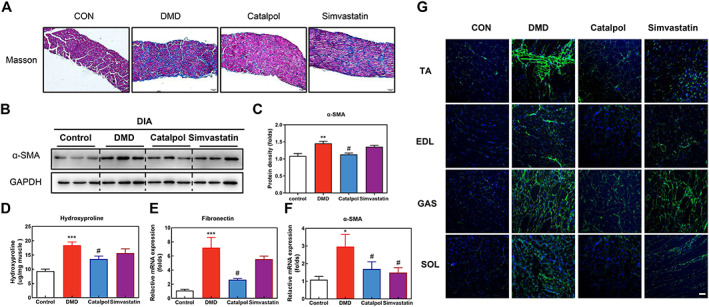
Catalpol minimizes diaphragm and tibialis anterior muscle fibrosis in *mdx* mice. (A) Representative Masson's trichrome stained images of DIA muscle sections. (B) Western blotting of α‐smooth muscle actin in DIA muscles and (C) the pooled values for each group. (D) Collagen I level in homogenized DIA muscles were determined by the hydroxyproline. (E, F) Real‐time quantitative polymerase chain reaction analysis of fibronectin and α‐smooth muscle actin expressions in TA muscles. (G) Representative images showing connective tissue levels in TA, EDL, GAS, and SOL muscles by fibronectin (green) immunostaining, and nuclei are stained with DAPI (blue). Scale bar, 50 μm. Comparisons were carried out using one‐way ANOVA with the Tukey–Kramer *post hoc* test. All data are shown as means ± SD (*n* = 12). ^*^ *P <* 0.05, ^**^ *P <* 0.01, ^***^ *P <* 0.001 vs. control (CON); ^###^ *P <* 0.001 vs. *mdx* mice (Duchenne muscular dystrophy).

The replacement of muscle fibres with fibrotic connective tissue is a major cause of muscle degeneration in DMD; therefore, fibrosis prevention is an important therapeutic approach.^27^ First, we evaluated fibrosis in the DIA muscle with Masson's trichrome staining, which was reduced in the catalpol‐treated *mdx* mice (*Figure* 2A). This effect was stronger than that of the simvastatin treatment. The western blotting results were consistent with the Masson's trichrome staining findings of anti‐fibrotic effects in *mdx* muscle induced by catalpol treatment (*P* < 0.05, *Figure* 2B and 2C). Next, we evaluated fibrosis in the TA muscle with a hydroxyproline assay, which also revealed a 29% reduction in fibrosis in catalpol‐treated *mdx* mice (*P* < 0.05, *Figure* 2D). The total fibronectin and α‐smooth muscle actin (SMA) mRNA levels in the TA muscle, quantified by real‐time quantitative polymerase chain reaction, also indicated an obvious reduction in fibrosis in the catalpol‐treated *mdx* mice (*P* < 0.05, *Figure* 2E and 2F). We further evaluated fibrosis of the TA, EDL, GAS, and SOL muscles using fibronectin immunofluorescence, which indicated a dramatic reduction in the fibronectin level in catalpol‐treated *mdx* mice (*Figure* 2G). Overall, these results indicated that catalpol has an anti‐fibrotic effect in *mdx* mice.

### Dystrophin deficiency impairs the differentiation of isolated primary myoblasts from mdx mice

**FIGURE 3:**
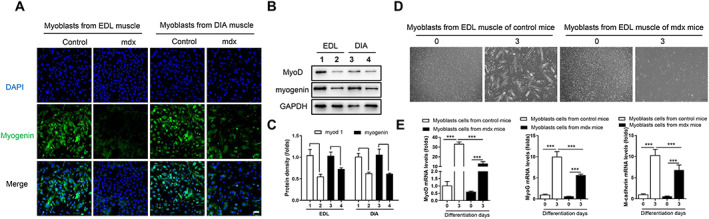
Dystrophin deficiency impairs the differentiation of isolated primary myoblasts from *mdx* mice. Isolated primary myoblasts from EDL and DIA muscle of control or *mdx* mice were cultured in differentiation medium for 3 days. (A) Myoblasts were stained with an anti‐myogenin (green) antibody and with DAPI staining of nuclei (blue). Scale bar, 50 μm. (B, C) Western blot of MyoD and myogenin protein levels (1: myoblasts from EDL muscle of control mice; 2: myoblasts from EDL muscle of *mdx* mice; 3: myoblasts from DIA muscle of control mice; and 4: myoblasts from DIA muscle of *mdx* mice). (D) Isolated primary myoblasts from EDL muscle of control or mdx mice, which were then differentiated for 0 or 3 days. (E) Real‐time quantitative polymerase chain reaction analysis showed the expressions of MyoD, MyoG, and M‐cadherin in primary myoblasts after differentiation for 0 or 3 days. Comparisons were carried out using two‐way ANOVA with the Tukey–Kramer *post hoc* test. All data are shown as means ± SD (*n* = 6). ^**^ *P* < 0.01, ^***^ *P* < 0.001 vs. controls.

The *mdx* mice had hypertrophic TA, EDL, GAS, and SOL muscles at 12 weeks of age, which we postulated was a compensatory protective effect on muscle regeneration after acute injury. To determine whether the compensatory protective effects on muscle regeneration were associated with dystrophin deficiency *in vivo*, we examined the differentiation ability of isolated myoblasts. First, we evaluated myoblast differentiation using myogenin immunofluorescence, which showed marked attenuation of the myogenin level in myoblasts from the EDL and DIA muscles of *mdx* mice compared with those from control mice (*Figure* 3A). These results were supported by those of western blot analysis, which showed obvious decreases in MyoD and myogenin levels in myoblasts from the EDL and DIA muscles of *mdx* mice compared with those from control mice (*P* < 0.001, *Figure* 3B and 3C). To confirm that dystrophin deficiency impairs myoblast differentiation, we treated the isolated primary myoblasts with differentiation medium for 3 days and found myotube formation only in myoblasts from control mice (*Figure* 3D). Quantification of the MyoD, MyoG, and M‐cadherin mRNA levels in isolated primary myoblasts after differentiation for 0 and 3 days showed a dramatic difference in differentiation at Day 3. Notably, MyoD, MyoG, and M‐cadherin gene expression in myoblasts from *mdx* mice was lesser than that in myoblasts from control mice (*P* < 0.001, *Figure* 3E). These findings support the notion that dystrophin deficiency impairs myoblast differentiation.

**FIGURE 4:**
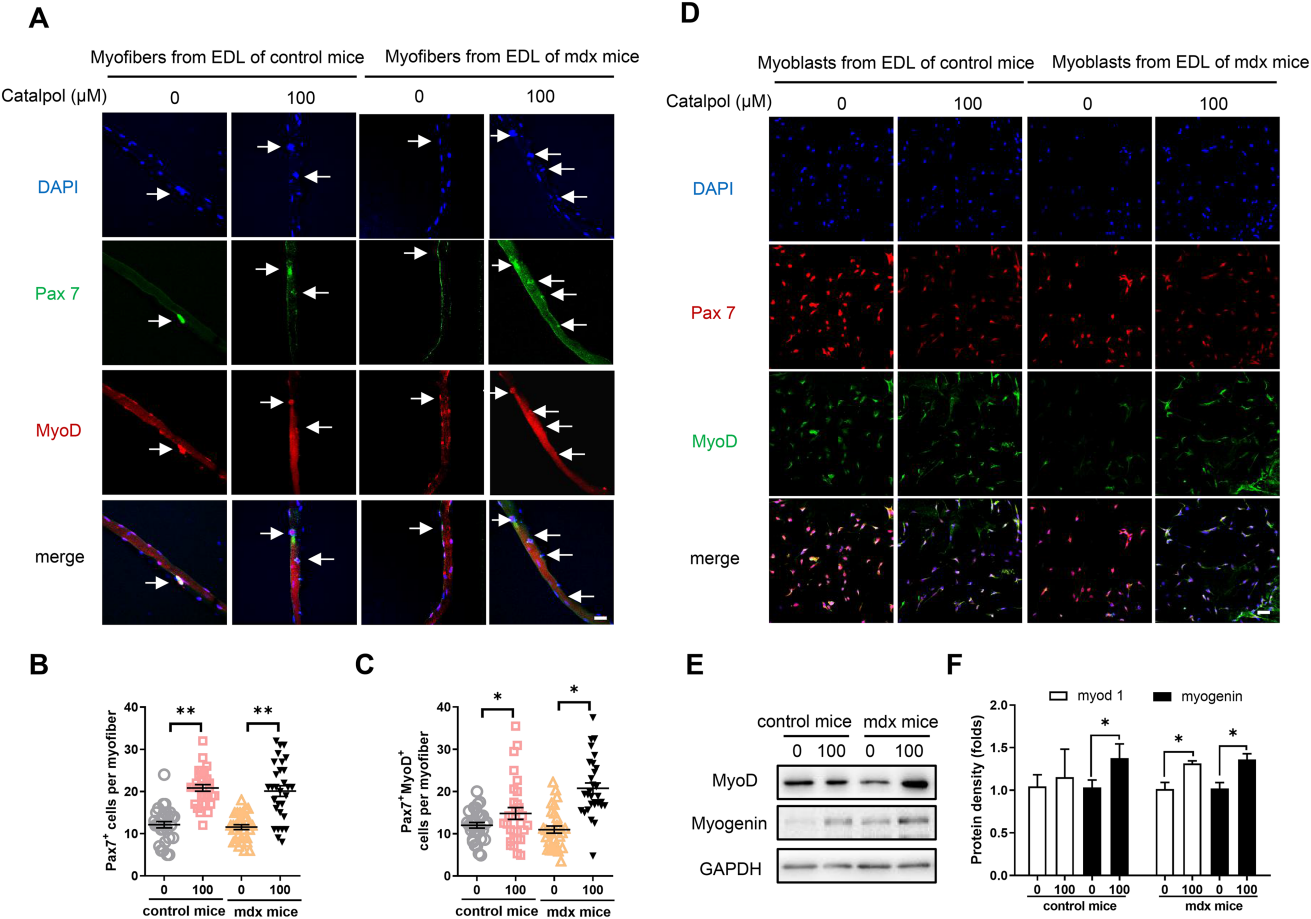
Catalpol enhanced the differentiation of primary myoblasts from EDL muscle of control or *mdx* mice. (A) Myofibres were stained with an anti‐Pax7 (green) antibody, anti‐MyoD (red), and with DAPI staining of nuclei (blue). Scale bar, 50 μm. (B) the number of Pax7^+^ cells per myofibres and (C) the number of Pax7^+^ MyoD^+^ cells per myofibres for each group. Isolated primary myoblasts from EDL muscle of control or mdx mice were treated with catalpol for 24 h. (D) Myoblasts were stained with an anti‐Pax7 (red) antibody, anti‐MyoD (green), and with DAPI staining of nuclei (blue). Scale bar, 50 μm. (E, F) Western blot of MyoD and myogenin protein levels. Comparisons were carried out using two‐way ANOVA with the Tukey–Kramer *post hoc* test. All data are shown as means ± SD (*n* = 6). ^*^ *P <* 0.05 vs. controls.

Previously, we found that catalpol improves skeletal muscle function by enhancing MyoD‐mediated/MyoG‐mediated myogenesis.^18^ We initially used isolated primary myofibres from *mdx* and control mice to study the activation of skeletal muscle satellites after catalpol treatment. We found that catalpol increased the number of Pax7^+^ cells (*P* < 0.01, *Figure* 4A and 4B) and Pax7^+^ MyoD^+^ cells (*P* < 0.05, *Figure* 4A and 4C) in myofibres from *mdx* and control mice, which indicated that catalpol increases and activates skeletal muscle satellites in myofibres. To examine whether catalpol enhances differentiation, we measured the Pax7, MyoD, and myogenin proteins after catalpol treatment in isolated primary myoblasts from *mdx* and control mice. This analysis showed that catalpol increased the MyoD and myogenin protein levels (*P* < 0.05, *Figure* 4D–F) compared with those in controls, implying that catalpol enhances the activation and differentiation of skeletal muscle satellite cells.

### Catalpol inhibited the phosphorylation of TAK1 by binding to the TAK1 protein

**FIGURE 5:**
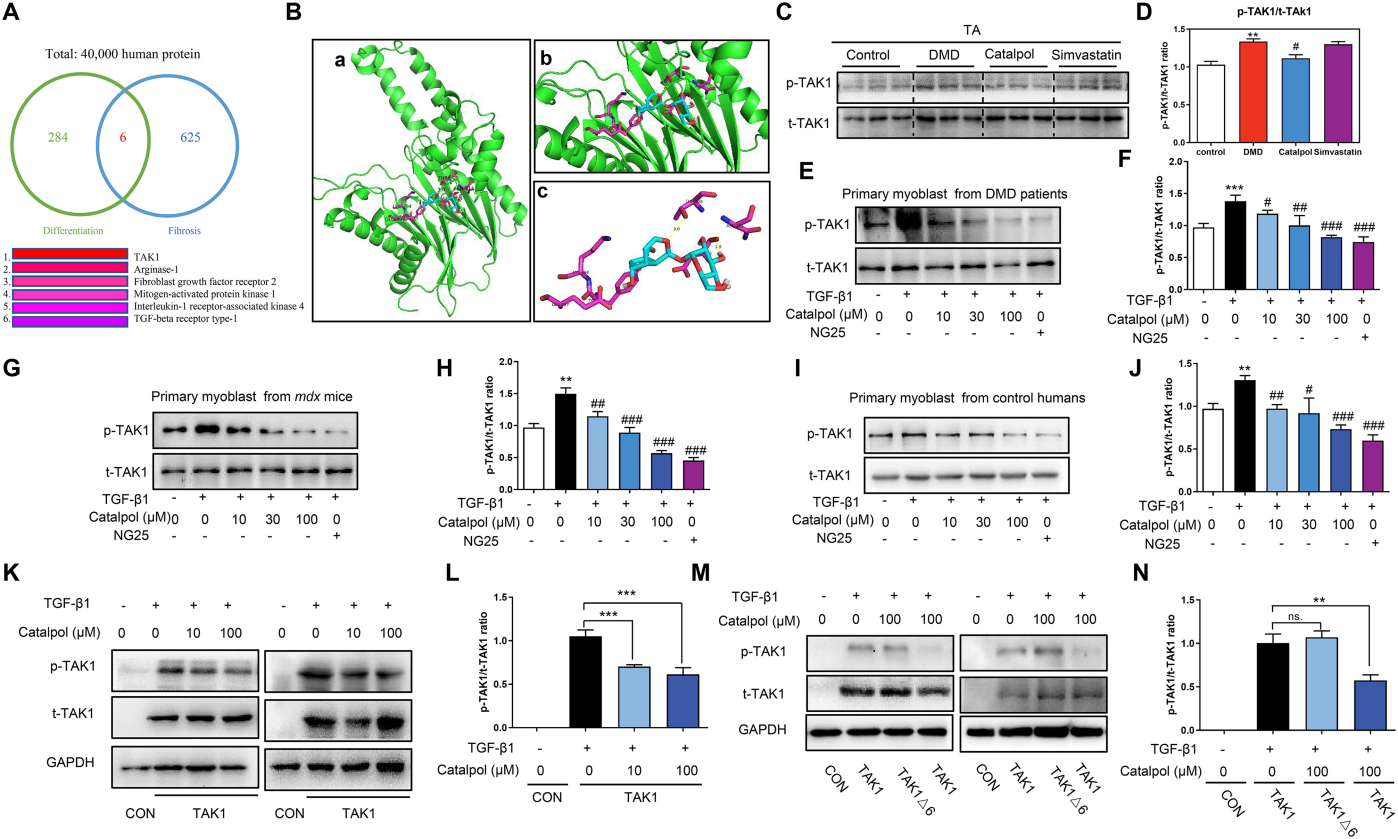
Catalpol treatment inhibited the phosphorylation of TAK1 in muscle tissue and primary myoblasts of mdx mice. (A) Molecular modelling studies of catalpol and 40 000 human proteins. Around 284 proteins are related with regulating myoblast differentiation; 625 proteins are related with regulating fibrosis; only six protein are related with regulating both myoblast differentiation and fibrosis. (B) Poses of catalpol are shown in the genomic site of TAK1 (PDB code: O43318), and the binding pocket of catalpol included Asp‐206, Thr‐208, Asn‐211, Glu‐297, Lys‐294, and Tyr‐293. (C) Western blot of p‐TAK1 and t‐TAK1 protein levels in TA muscle and (D) the pooled values of p‐TAK1/t‐TAK1 for each group. Comparisons were carried out using one‐way ANOVA with the Tukey–Kramer *post hoc* test. Values are means ± SD (*n* = 6), ^**^ *P <* 0.01 vs. the control; ^#^ *P <* 0.05 vs. the DMD group. Isolated primary myoblasts were incubated in serum‐free media with 5 ng/ml TGF‐β1 and containing 10, 30, and 100 μM catalpol or 10 μM NG25 for 24 h. Western blot of p‐TAK1 and t‐TAK1 protein levels in primary myoblasts from (E, F) Duchenne muscular dystrophy patients, (G, H) *mdx* mice, (I, J) control humans, and the pooled values of p‐TAK1/t‐TAK1 for each group. Comparisons were carried out using one‐way ANOVA with the Tukey–Kramer *post hoc* test. All data are shown as means ± SD (*n* = 3), ^**^ *P <* 0.01, ^***^ *P <* 0.001 vs. the control; ^#^ *p <* 0.05, ^##^ *p <* 0.01, ^###^ *p <* 0.001 vs. the TGF‐β1 group. **(**K, L**)** Human embryonic kidney 293 T cells were transduced with TAK1 DNA plasmid for 24 h and then incubation with 5 ng/mL TGF‐β1 and containing catalpol (0, 10, and 100 μM) for 24 h. Western blot of p‐TAK1 and t‐TAK1 protein levels and the pooled values for each group. Human embryonic kidney 293 T cells were transduced with TAK1 DNA plasmid or mutational TAK1△6 (Asp‐206, Thr‐208, Asn‐211, Glu‐297, Lys‐294, and Tyr‐293) DNA plasmid for 24 h and then incubation with 5 ng/ml TGF‐β1 and containing catalpol (0 and 100 μM) for 24 h. (M, N) Western blot of p‐TAK1 and t‐TAK1 protein levels and the pooled values for each group. ^**^ *p <* 0.01 vs. the control.

To study the molecular mechanism of the anti‐fibrotic and enhanced differentiation effects of catalpol, we performed computer modelling docking studies of the high‐resolution crystal structure of human proteins with catalpol. We found that 284 proteins were involved in the regulation of myoblast differentiation, and 625 proteins were involved in the regulation of fibrosis, with only six proteins involved in the regulation of both processes. TAK1 is a potential target regulating myoblast differentiation and fibrosis (*Figure* 5A). To examine the molecular interactions between catalpol and TAK1, we analysed the high‐resolution crystal structure of human TAK1 in complex with catalpol. We found that the catalpol‐binding pocket was composed of Asp‐206, Thr‐208, Asn‐211, Glu‐297, Lys‐294, and Tyr‐293 (*Figure* 5B). We estimated that the binding energies of catalpol and NG25 to TAK1 were −7.5 and −9.6 kcal/mol, respectively. Previous studies have shown that TAK1 (Thr‐184/187) is activated in inflammatory and fibrotic tissue.^28^, ^29^ To examine the effect of catalpol on the phosphorylation of TAK1, we first determined the p‐TAK1 and t‐TAK1 protein levels *in vivo*. The p‐TAK1/t‐TAK1 protein ratio was increased in *mdx* mice compared with control mice (*P* < 0.01, *Figure* 5C and 5D)). In addition, catalpol, but not simvastatin, treatment eliminated p‐TAK1 overexpression in the TA muscle of *mdx* mice (*P* < 0.05, *Figure* 5C and 5D). To confirm the effects of catalpol on TAK1, we determined the p‐TAK1/t‐TAK1 ratios in isolated primary myoblasts from patients with DMD, *mdx* mice, and control humans. We found that TGF‐β1 increased p‐TAK1 expression and that catalpol reduced the p‐TAK1/t‐TAK1 ratio in a dose‐dependent manner (*P* < 0.05, *Figure* 5E–J). These results suggest that catalpol can bind to TAK1 and suppress its phosphorylation.

To determine the position of catalpol binding to TAK1, we transiently transfected HEK293T cells with TAK1 and mutated TAK1 expression vectors. Next, we treated the cells with different concentrations of catalpol and measured p‐TAK1 protein levels. First, t‐TAK1 was overexpressed after transient transfection of HEK293T cells with TAK1 expression vector, and TGF‐β1 increased the expression of p‐TAK1. Moreover, the increase in the p‐TAK1 protein level was reversed by treatment with catalpol (*P* < 0.001, *Figure* 5K and 5L). The mutated TAK1 expression vector was designed and produced by GenScript Bio by replacing Asp‐206, Thr‐208, Asn‐211, Glu‐297, Lys‐294, and Tyr‐293 in the TAK1 expression vector with Ala (△6). Western blotting showed that the effect of catalpol on p‐TAK1 was eliminated with the mutated TAK1 expression vector (*Figure* 5M and 5N). These data suggest that catalpol suppresses the phosphorylation of TAK1 by binding TAK1 and that the binding position involves Asp‐206, Thr‐208, Asn‐211, Glu‐297, Lys‐294, and Tyr‐293.

### Catalpol alleviated fibrosis by preventing the trans‐differentiation of myoblasts into myofibroblasts

**FIGURE 6:**
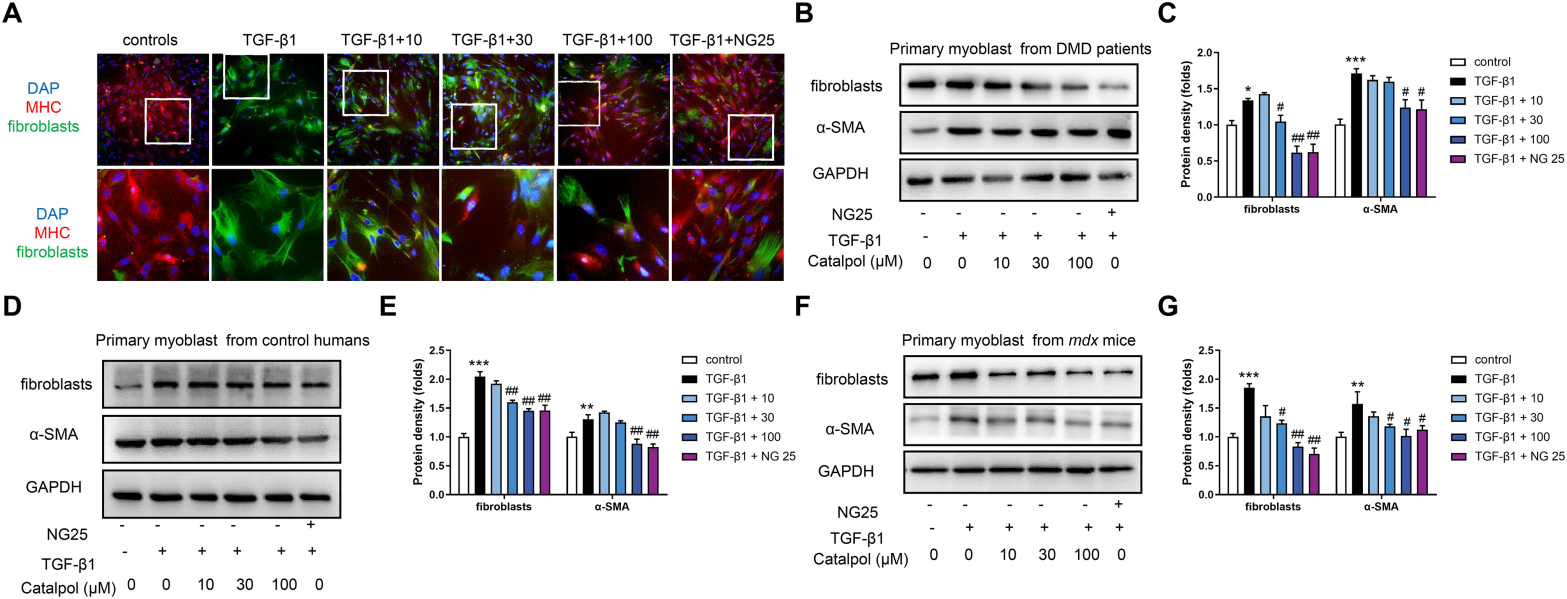
Catalpol inhibits TAK1‐meidated trans‐differentiation in primary myoblasts. (A) Representative images showing trans‐differentiation of primary myoblasts using myosin heavy chain and fibroblast antibodies. Isolated primary myoblasts were incubated in serum‐free media with 5 ng/mL TGF‐β1 and containing 10, 30, and 100 μM catalpol or 10 μM NG25 for 24 h. Western blot of fibroblast and α‐smooth muscle actin protein levels in primary myoblasts from (B, C) Duchenne muscular dystrophy patients, (D, E) control humans, (F, G) *mdx* mice, and the pooled values for each group. Comparisons were carried out using one‐way ANOVA with the Tukey–Kramer *post hoc* test. Values are means ± SD (*n* = 3), ^*^ *P <* 0.05, ^**^ *P <* 0.01, ^***^ *P <* 0.001 vs. the control; ^#^ *P <* 0.05, ^##^ *P <* 0.01 vs. the TGF‐β1 group.

A recent study showed that TGF‐β1/TAK1 activation plays a vital role in fibrosis in diverse diseases,^30^, ^31^, ^32^ but the relationship of TGF‐β1/TAK1 to muscle fibrosis in DMD has not been elucidated. Thus, we explored whether the anti‐fibrotic effect of catalpol was regulated via TGF‐β1/TAK1. Myofibroblasts form a unique group of smooth muscle‐like fibroblasts that play vital roles in inflammation and fibrosis.^32^ Myofibroblasts secrete inflammatory cytokines, growth factors, and extracellular matrix protein in most tissues. We used primary myoblasts from DMD to examine whether TGF‐β1 induces the trans‐differentiation of myoblasts into myofibroblasts. The treatment of myoblasts with TGF‐β1 (5 ng/mL) caused a marked increase in fibroblast and a significant reduction in the expression of the MHC, a known myogenic marker. Furthermore, catalpol and NG25 (a TAK1 inhibitor) offset the effects of TGF‐β1 on myoblasts (*Figure* 6A). Myofibroblasts express (α‐SMA) and promote fibrosis. Western blotting showed that TGF‐β1 increased fibroblast and α‐SMA expression in primary myoblasts from patients with DMD. As expected, catalpol and NG25 reduced the levels of fibroblast and α‐SMA (*Figure* 6B and 6C). Similar suppressive effects of catalpol and NG25 on fibroblast and α‐SMA expression were also observed in primary myoblasts from control humans (*Figure* 6D and 6E) and *mdx* mice (*Figure* 6F and 6G). Together, these data support the view that catalpol alleviates fibrosis by preventing trans‐differentiation of myoblasts into myofibroblasts.

### Catalpol enhanced myogenesis by preventing the trans‐differentiation of myoblasts into myofibroblasts

**FIGURE 7:**
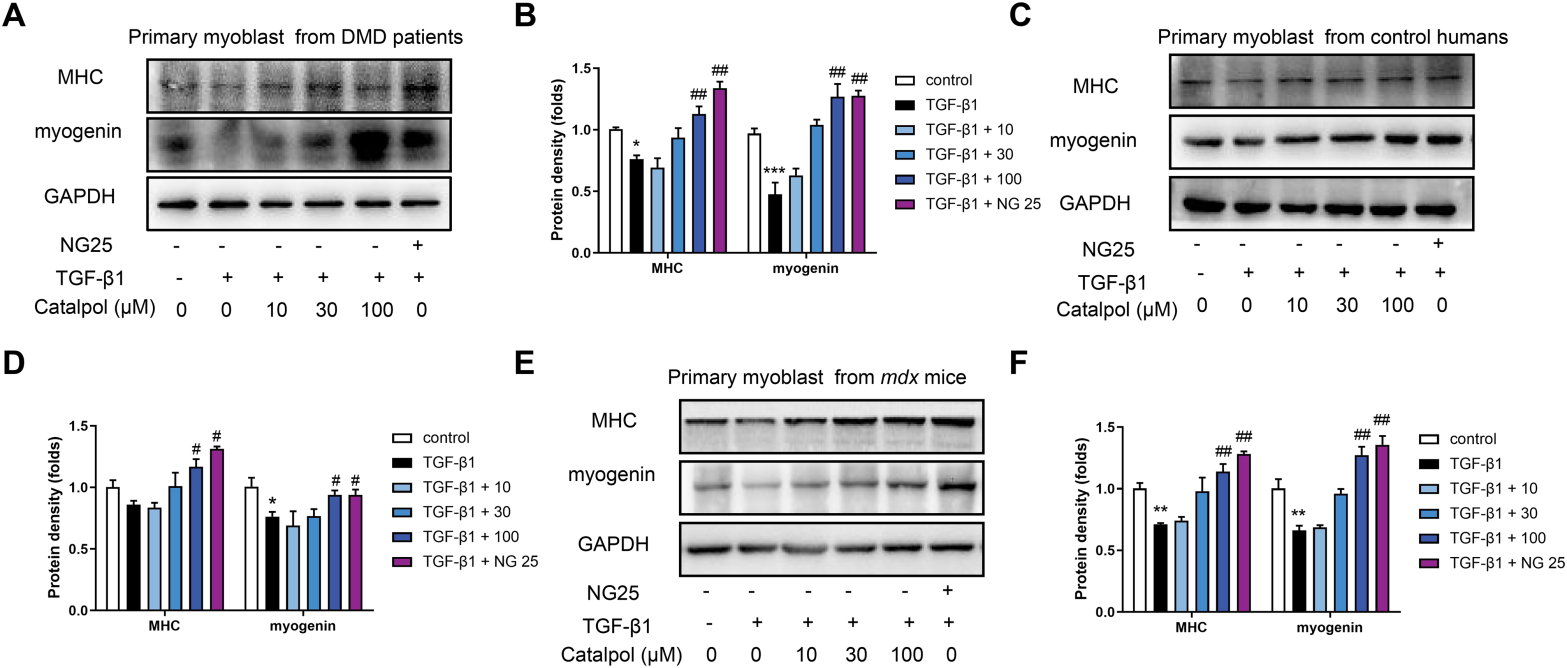
Catalpol blunts TGF‐β1 induced‐differentiation inhibition in primary myoblasts. Isolated primary myoblasts were incubated in serum‐free media with 5 ng/ml TGF‐β1 and containing 10, 30, and 100 μM catalpol or 10 μM NG25 for 24 h. Western blot of fibroblast, myosin heavy chain and myogenin protein levels in primary myoblasts from Duchenne muscular dystrophy patients (A, B), control humans (C, D), and *mdx* mice (E, F) and then the pooled values for each group. Comparisons were carried out using one‐way ANOVA with the Tukey–Kramer *post hoc* test. All data are shown as means ± SD (*n* = 3), ^*^ *P <* 0.05, ^**^ *P <* 0.01, ^***^ *P <* 0.001 vs. the control; ^#^ *P <* 0.05, ^##^ *P <* 0.01 vs. the TGF‐β1 group.

Interestingly, recent studies revealed that TGF‐β1 induces the trans‐differentiation of myoblasts into myofibroblasts^13^, ^33^ and inhibits myogenesis in skeletal muscle.^23^ In addition, the replacement of muscle fibres by fibrotic connective tissue is a major cause of muscle wasting in DMD. Therefore, we postulated that catalpol enhanced myogenesis by preventing the TGF‐β1‐induced over‐trans‐differentiation of myoblasts into myofibroblasts. Western blotting analysis indicated that the MHC and myogenin levels decreased dramatically after TGF‐β1 treatment of primary myoblasts from patients with DMD. Notably, catalpol and NG25 attenuated the inhibitory effect of TGF‐β1 on myogenesis, as shown by increased MHC and myogenin levels (*Figure* 7A and 7B). These results were also seen with primary myoblasts from control humans (*Figure* 7C and 7D) and *mdx* mice (*Figure* 7E and 7F). These findings demonstrate that catalpol enhanced myogenesis by preventing TGF‐β1–induced trans‐differentiation.

### TGF‐β1‐induced trans‐differentiation of myoblasts into myofibroblasts required the up‐regulation of TAK1

**FIGURE 8:**
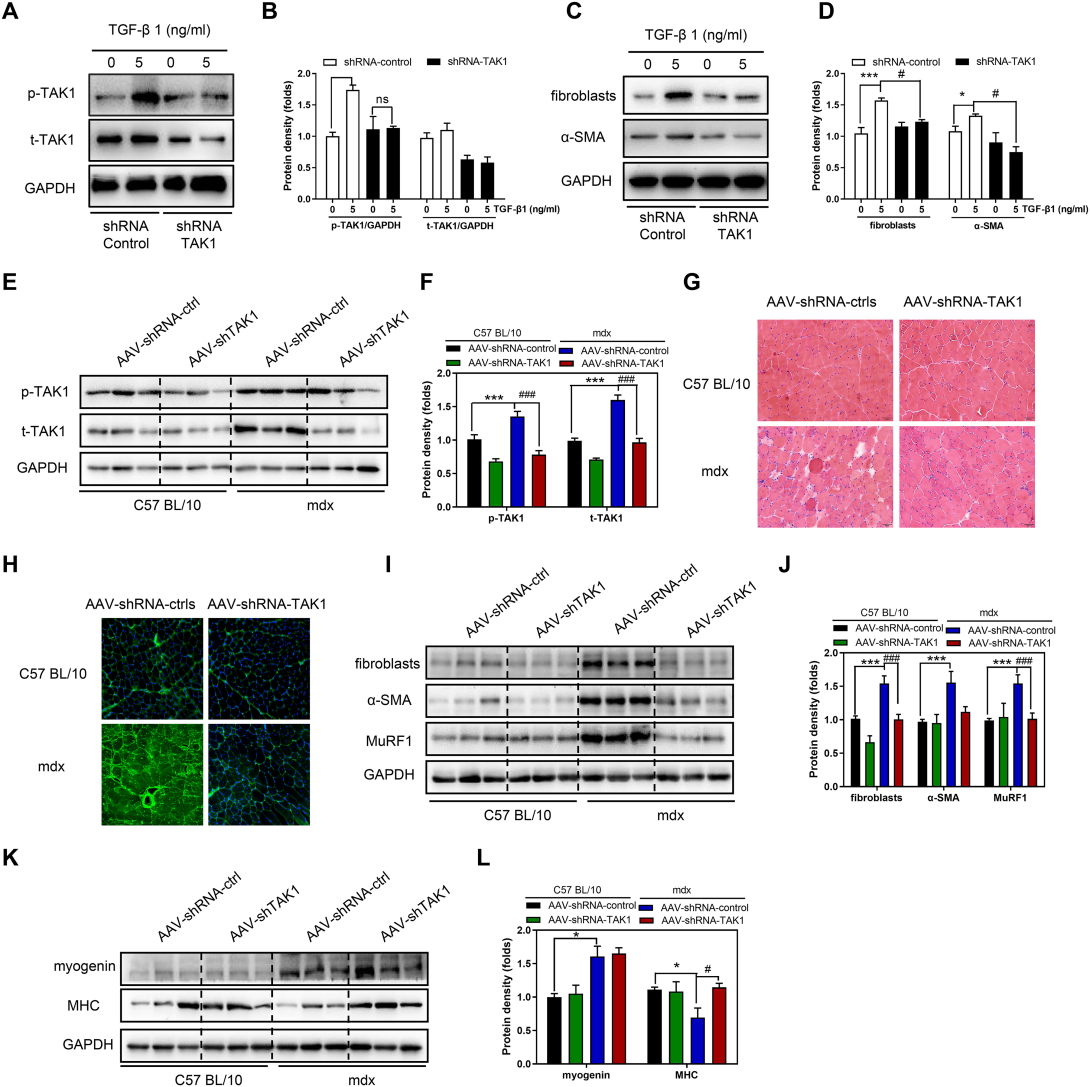
TAK1 knockdown protects against fibrosis and enhances myoblasts differentiation in *mdx* mice. Primary myoblasts were transduced with Ad. shTAK1 for 24 h followed by incubation in DM containing 0 or 5 ng/ml TGF‐β1. (A, B) Western blot of p‐TAK1 and t‐TAK1 protein levels in primary myoblasts and the pooled values for each group. (C, D) Western blot of fibroblast and α‐smooth muscle actin protein levels in primary myoblasts and the pooled values for each group. (E, F) AAV‐shRNA‐control and AAV‐shRNA‐TAK1 were injected intramuscularly in the right and left TA muscles, respectively. Western blot of p‐TAK1 and t‐TAK1 protein levels in TA muscle of *mdx* mice and control mice. (G) Representative haematoxylin and eosin‐stained images of TA sections from control mice (a: AAV‐shRNA‐ctrl; b: AAV‐shTAK1) and *mdx* mice (c: AAV‐shRNA‐ctrl; d: AAV‐shTAK1). (H) Representative images showing fibrosis of TA sections from control mice (a: AAV‐shRNA‐ctrl; b: AAV‐shTAK1) and *mdx* mice (c: AAV‐shRNA‐ctrl; d: AAV‐shTAK1) using fibronectin antibodies (green) and nuclei are stained with DAPI (blue). (I, J) Western blot of fibroblast, α‐SMA, and MuRF1 protein levels in the TA. (K, L) Western blot of myogenin and myosin heavy chain protein levels in the TA. Comparisons were carried out using two‐way ANOVA with the Tukey–Kramer *post hoc* test. All data are shown as means ± SD (*n* = 6), ^**^ *P <* 0.01 vs. the control; ^#^ *P <* 0.05 vs. the Duchenne muscular dystrophy group.

To explore the importance of TAK1 in TGF‐β1‐induced myofibroblast trans‐differentiation, we examined whether the inhibition of TAK1 eliminated the effect of TGF‐β1 on primary myoblasts. First, we used shRNA‐TAK1 to suppress TAK1 expression in primary myoblasts from patients with DMD. The effect of TGF‐β1 on TAK1 activation was eliminated by treatment with shRNA‐TAK1 (*P* < 0.05, *Figure* 8A and 8B). The trans‐differentiation of myofibroblasts induced by TGF‐β1 was also eliminated by shRNA‐TAK1 treatment, suggesting that this trans‐differentiation requires TAK1 up‐regulation (*Figure* 8C and 8D). Next, we examined the importance of TAK1 in TGF‐β1‐induced myofibroblast trans‐differentiation in *mdx* mice. AAV‐shRNA‐control and AAV‐shRNA‐TAK1 were injected intramuscularly in the right and left TA muscles, respectively, of *mdx* and control mice. p‐TAK and t‐TAK1 expression increased initially in the AAV‐shRNA‐control *mdx* mice, but AAV‐shTAK1 eliminated this effect on up‐regulation of TAK1 (*P* < 0.001, *Figure* 8E and 8F). Histological assessment and fibronectin immunofluorescence staining of the TA muscle showed significantly decreased inflammation and fibrosis in the AAV‐shRNA‐TAK1 *mdx* mice compared with the AAV‐shRNA‐control *mdx* mice (*Figure* 8G and 8H). These data suggest that fibrosis caused by the up‐regulation of TAK1 is alleviated by the inhibition of TAK1 in dystrophic muscle. We also examined whether the anti‐fibrotic effect was caused by inhibition of the trans‐differentiation of myoblasts into myofibroblasts. The levels of fibroblast and α‐SMA were increased in the AAV‐shRNA‐control *mdx* mice compared with the AAV‐shRNA‐control control mice. However, the overexpression of fibroblast and α‐SMA was decreased in the AAV‐shRNA‐TAK1 *mdx* mice (*P* < 0.001, *Figure* 8I and 8J). In addition, the MHC level was reduced in the AAV‐shRNA‐control *mdx* mice, but this effect was reversed by AAV‐shRNA‐TAK1 in *mdx* mice (*P* < 0.05, *Figure* 8K and 8L). Overall, these findings support the postulations that TAK1 up‐regulation is required for the trans‐differentiation of myoblasts into myofibroblasts and that the inhibition of TAK1 could alleviate fibrosis and enhance myogenesis in *mdx* mice.

## Discussion

**FIGURE 9:**
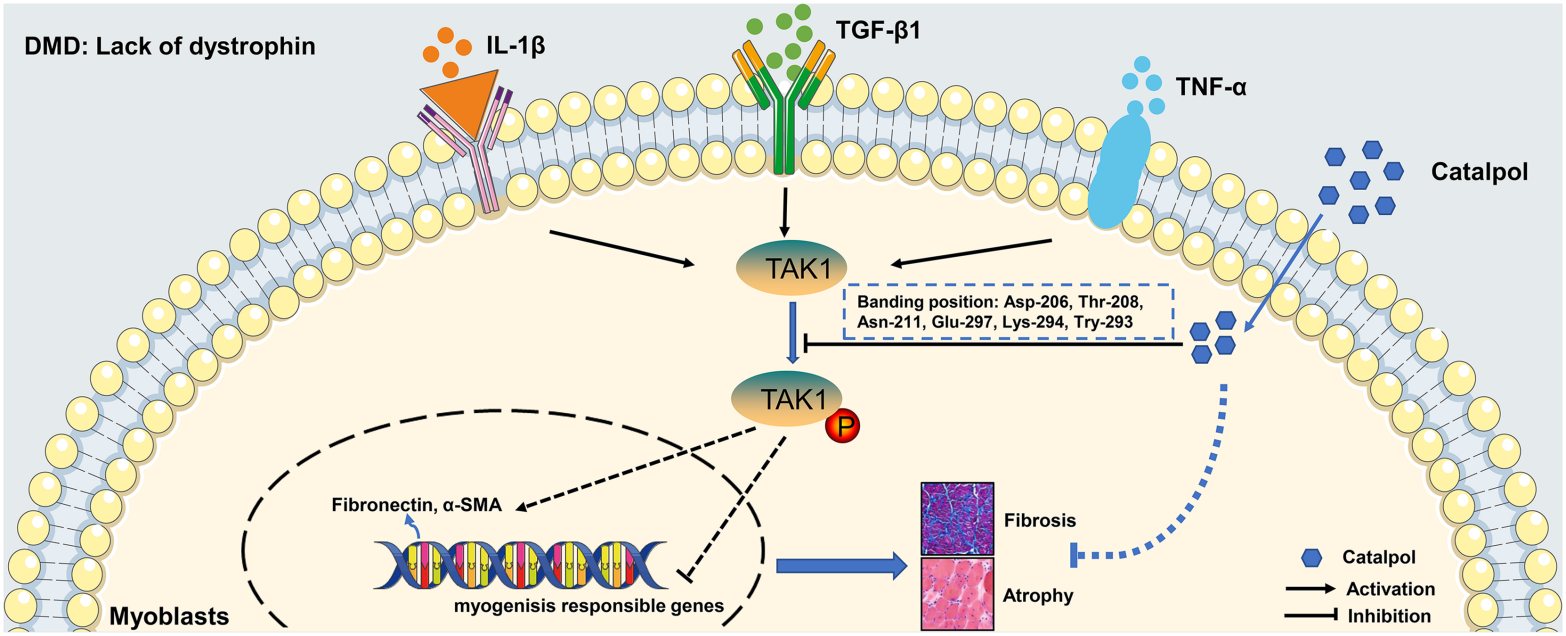
Schematic diagram depicting the possible mechanism of catalpol for Duchenne muscular dystrophy treatment by binding with TAK1 in skeletal muscle. TGF‐β1, IL‐1β, and TNF‐α are secreted by macrophage in dystrophic muscle, which result in TAK1 phosphorylation. TAK1 phosphorylation aggravates muscle fibrosis and muscle degeneration in DMD. Catalpol improves the dystrophic muscle function by competitive binding with TAK1 to avoid over‐phosphorylation of TAK1, which subsequently results in reduced fibrosis and enhancement of myoblasts differentiation. Moreover, the binding position of TAK1 may be Asp‐206, Thr‐208, Asn‐211, Glu‐297, Lys‐294, and Tyr‐293.

A recent study showed that catalpol prevents denervated muscular atrophy.^34^ In addition, we previously reported that catalpol improves muscle function by activating MyoD/MyoG‐mediated myogenesis.^18^ Consistent with these findings, we showed in this study that catalpol significantly restored muscular function via its anti‐fibrotic effects and enhanced myogenesis in *mdx* mice. Moreover, we identified the direct target of catalpol: catalpol inhibits the phosphorylation of TAK1 by binding to TAK1, possibly at Asp‐206, Thr‐208, Asn‐211, Glu‐297, Lys‐294, and Tyr‐293. In human and mouse primary myoblasts, we found that the TGF‐β1‐induced trans‐differentiation of myoblasts into myofibroblasts required TAK1 up‐regulation. Catalpol prevents the over‐trans‐differentiation of myoblasts into myofibroblasts by suppressing the phosphorylation of TAK1, which has anti‐fibrotic effects and restores myogenesis in skeletal muscle (*Figure* 9). Overall, the down‐regulation of TAK1‐mediated over‐trans‐differentiation in myoblasts plays a vital role in the anti‐muscle atrophy effect of catalpol.

In patients with DMD and *mdx* mice, muscle degeneration results from several pathogenic processes, and oxidative stress, fibrosis, and chronic inflammation have major effects on the functional impairment of muscle and on disease progression.^35^, ^36^ Our study reveals for the first time that the treatment of *mdx* mice with catalpol reduces fibrosis and inflammation, major pathogenic pathways that mediate muscle degeneration and functional impairment in DMD. These pathological changes translated into improved skeletal muscle function, as reflected in grip strength *in vivo* and specific force production and recovery *in vitro*. Most importantly, we observed reductions in CK and LDH, widely used clinical markers of muscle damage. Thus, catalpol has a positive effect in DMD treatment. Simvastatin, which is used widely for the reduction of total cholesterol, has been reported to dramatically reduce inflammation, fibrosis, and oxidative stress in *mdx* mice.^25^ In this study, we found that simvastatin had a stronger anti‐inflammatory effect, but a weaker anti‐fibrotic effect, than catalpol (*Figure* S1). A previous study showed that the anti‐inflammatory effect of simvastatin was accompanied by autophagy activation^37^; here, we showed that the anti‐fibrotic effect of catalpol was caused by the prevention of over‐trans‐differentiation of myoblasts into myofibroblasts.

Increasing reports suggest that TGF‐β1/TAK1 signalling plays critical roles in extracellular matrix production and the pathogenesis of fibrosis.^30^, ^38^ TAK1 mediates fibrosis by increasing the expression of type I collagen and fibronectin.^39^, ^40^ In this study, we found that fibrosis was accompanied by TAK1 activation in *mdx* mice. However, the fibrosis was prevented by treatment with catalpol or AAV‐shTAK1. Furthermore, we found that TGF‐β1‐induced fibrosis required the up‐regulation of TAK1 in human and mouse primary myoblasts. Catalpol and NG25 alleviated fibrosis by inhibiting the phosphorylation of TAK1, as indicated by decreased α‐SMA and fibronectin levels. Overall, our study provides evidence that the activation of TAK1 plays a critical role in fibrosis in DMD and that the prevention of TAK1 phosphorylation may be a new anti‐fibrosis approach.

TAK1 plays important roles in satellite cell homoeostasis and function. It regulates skeletal muscle mass and mitochondrial function.^41^ The targeted knockout of TAK1 caused increased oxidative stress and dysfunctional regeneration in response to muscle injury.^14^ By contrast, many studies have shown that TAK1 activators inhibit myogenic differentiation,^42^, ^43^, ^44^ implying that TAK1 is a negative regulator of differentiation. Moreover, other studies have indicated that TAK1 overexpression can increase proliferation and reduce differentiation.^23^, ^39^ Despite the growing controversy, TAK1 is related to myogenesis. In this study, we found that TAK1 was activated and that its phosphorylation was increased in *mdx* mice (*Figure* 5B and 5C), which was accompanied by increased centrally nucleated (regenerating) myofibres (*Figure* 1I). In addition, an inhibitor of myogenic differentiation was found in primary myoblasts from *mdx* mice (*Figure* 3A–C), which was accompanied by TAK1 activation (*Figure* 5). Thus, the activation of TAK1 increased cell proliferation and inhibited differentiation, as reported previously.^8^ Most importantly, we found that the inhibition of myogenic differentiation induced by TGF‐β1 required the up‐regulation of TAK1 and that catalpol and NG25 treatments eliminated this effect in primary myoblasts, implicating TAK1 as a regulator of myoblast differentiation. Although TGF‐β1 inhibition has been reported to have a protective effect in DMD, the detailed mechanism seems unclear. The therapeutic effect of TAK1 inhibition may be stronger than that of TGF‐β1 inhibition in DMD. We will compare the effects of these two potential targets in further studies.

A previous study showed that TGF‐β1 induced the trans‐differentiation of myoblasts into myofibroblasts.^45^ In this study, we demonstrated that fibrosis and loss of myofibres were caused by the over‐trans‐differentiation of myoblasts into myofibroblasts in DMD. We showed that TGF‐β1 could induce the trans‐differentiation of myoblasts into myofibroblasts but that this process required the up‐regulation of TAK1. Knockdown of TAK1 by AAV‐shTAK1 and treatment with catalpol can alleviate fibrosis and improve skeletal muscle function, which may be related to prevention of the over‐trans‐differentiation of myoblasts into myofibroblasts. The current study demonstrated that catalpol suppressed the phosphorylation of TAK1 by binding to TAK1, possibly at Asp‐206, Thr‐208, Asn‐211, Glu‐297, Lys‐294, and Tyr‐293. Further studies will investigate the detailed target position of TAK1 and how to bind to that position.

Pharmacological anti‐inflammatory therapy remains a main treatment strategy for DMD and related neuromuscular diseases. However, other studies have shown that muscle regeneration requires inflammatory cytokines to activate muscle satellite cells following skeletal muscle injury.^46^, ^47^ Thus, inflammatory cytokines play important roles in muscle repair after injury. We found a significant increase in inflammatory cytokines in *mdx* mice compared with control mice, as reported previously.^48^ To control the pro‐fibrotic reaction induced by inflammatory cytokines, but not prevent muscle regeneration, we did not clear inflammatory cytokines directly. TAK1 is activated by IL‐1, TNF‐α, and TGF‐β1.^49^ Catalpol improves myoblast differentiation and alleviates fibrosis in *mdx* mice by inhibiting TAK1, which is a potential approach to DMD treatment.

In summary, our study provides evidence that increased fibrosis and decreased differentiation induced by the increased trans‐differentiation of myoblasts into myofibroblasts were caused by the overexpression of TAK1 in dystrophic muscle. Our study revealed the protective effects of catalpol on fibrosis and muscle weakness in *mdx* mice. Further studies are required to clarify the detailed mechanism by which TAK1 regulates the trans‐differentiation of myoblasts. Catalpol and possibly other TAK1 inhibitors have the potential to treat DMD.

## Funding

This work was supported by the grants from the Scholar of the 14th Batch of “Six Talents Peak” High‐level Talent Selection program (SWYY‐094); the Postgraduate Research Practice Innovation Program of Jiangsu Province (KYCX19‐0763); the “Double First‐Class” University project (CPU2018GY33); the National Natural Science Foundation of China (No. 81773827, 81573514), to J.Z.; the National Natural Science Foundation of China (No. 81773995), to Z.L.; the National Natural Science Foundation of China (No. 81873084, 81573690), to S.L.

## Author Contributions

Dengqiu Xu and Jingwei Jiang researched the data, contributed to discussions, and wrote the manuscript. Xihua Li and Lei Zhao collected and analysed clinical data. Zeren Sun, Xiaofei Huang, and Sijia Li collected and analysed data. Lixin Sun, Xin Huang, and Tao wang reviewed the manuscript. Zhenzhou Jiang conceived the experiments, researched the data, and edited/reviewed the manuscript. Luyong Zhang is the guarantor of this work and had full access to all the data in the study and takes responsibility for the integrity of the data and the accuracy of the data analysis.

## Conflict of interest

The authors declare that there are no conflicts of interest.

## Supporting information

Figure S1. Representative H&E‐stained images of TA, DIA and GAS sections from mice (magnification 200×). Representative images showing inflammation of TA, DIA and GAS sections using CD68 antibodies (green), laminin (red) and nuclei are stained with DAPI (blue). Scale bar, 50 μm.

Table S1. The primers used for Real‐time quantitative PCR (house mouse)
