# Supplementary figures and images for: A potential therapeutic effect of catalpol in Duchenne muscular dystrophy revealed by binding with TAK1
Source: J Cachexia Sarcopenia Muscle. 2020 Aug 31;11(5):1306–20. doi: 10.1002/jcsm.12581 (PMC7567147; doi:10.1002/jcsm.12581)

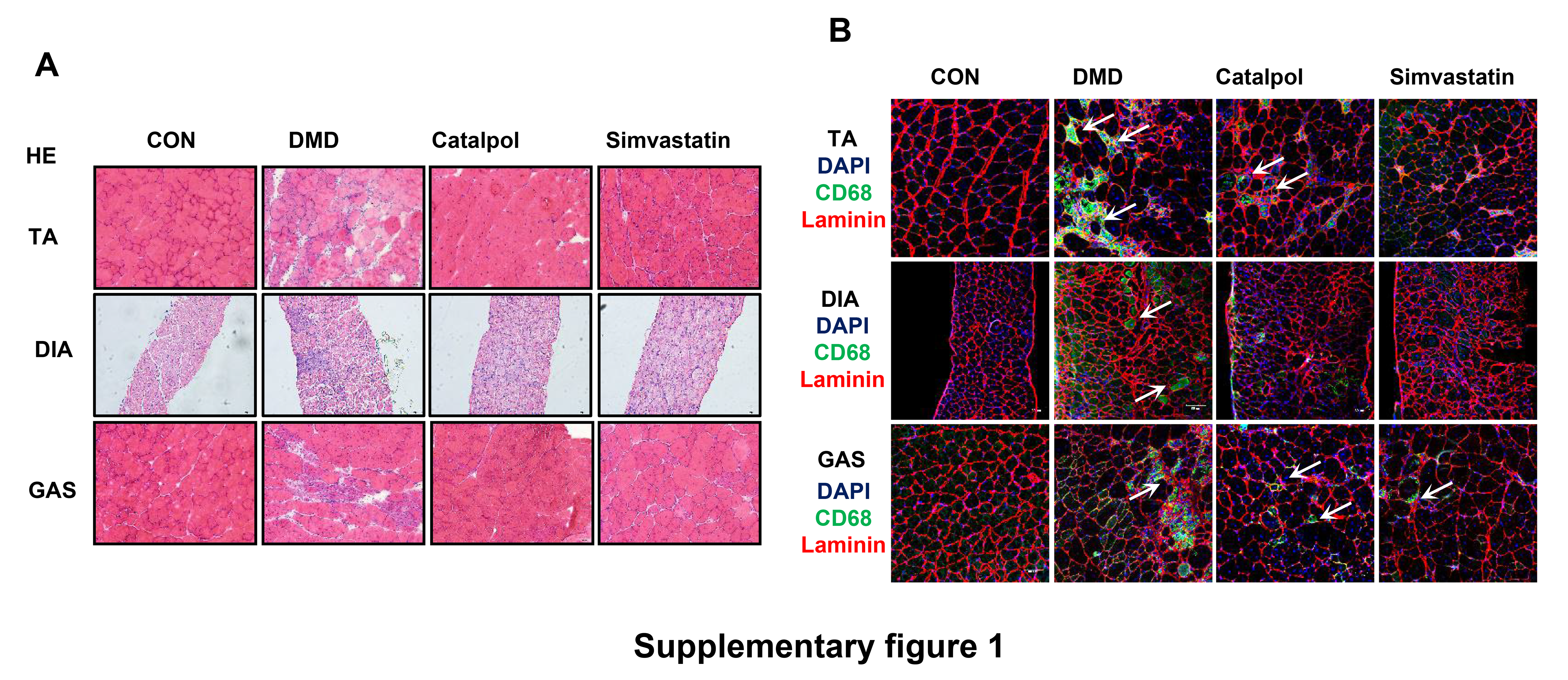

Supplement: Supplementary file 1 — Figure S1. Representative H&E‐stained images of TA, DIA and GAS sections from mice (magnification 200×). Representative images showing inflammation of TA, DIA and GAS sections using CD68 antibodies (green), laminin (red) and nuclei are stained with DAPI (blue). Scale bar, 50 μm. [file JCSM-11-1306-s001.tif]
