# Supplementary material for: A potential therapeutic effect of catalpol in Duchenne muscular dystrophy revealed by binding with TAK1
Source: J Cachexia Sarcopenia Muscle. 2020 Aug 31;11(5):1306–20. doi: 10.1002/jcsm.12581 (PMC7567147; doi:10.1002/jcsm.12581)
Supplement: Supplementary file 2 — Table S1. The primers used for Real‐time quantitative PCR (house mouse) [file JCSM-11-1306-s002.docx]

| Suppl. table 1. The primers used for Real-time quantitative PCR (house mouse) | | | |
| --- | --- | --- | --- |
| Gene |  | | NCBI |
| GAPDH | Forward | CCATTTGCAGTGGCAAAG | NC_000072.6 |
|  | Reverse | CACCCCATTTGATGTTAGTG |  |
| MyoD | Forward | GCCGCCTGAGCAAAGTGAATG | NC_000073.6 |
|  | Reverse | CAGCGGTCCAGGTGCGTAGAAG |  |
| MyoG | Forward | GGTGTGTAAGAGGAAGTCTGTG | NC_000067.6 |
|  | Reverse | TAGGCGCTCAATGTACTGGAT |  |
| M-cadherin | Forward | ATGTGCCACAGCCACATCG | NC_000074.6 |
|  | Reverse | TCCATACATGCTCGCCAGC |  |
| Fibronectin | Forward | AAGGAGTTTCATCTGGCCCT | NC_000077.6 |
|  | Reverse | AGCAGGTCCTTGGAAACCTT |  |
| α-SMA | Forward | TCAGGGAGTAATGGTTGGAATG | NC_000085.6 |
|  | Reverse | GTGTCGGATGCTCTTCAGG |  |

| Suppl. table 2. The antibody used for Western blot analysis | | | |
| --- | --- | --- | --- |
| Primary antibody | Company | Cat: number | Dilution |
| Anti-myogenin | Santa Cruz | sc-52903 | 1:500 |
| Anti-α-SMA | Cell Signaling Technology | 19245 | 1:1000 |
| Anti-MyoD | Santa Cruz | sc-377460 | 1:500 |
| Anti-MHC | DSHB | MF 20 | 1:200 |
| Anti-MuRF1 | Santa Cruz | sc-398608 | 1:500 |
| Anti-phospho-TAK1 | Cell Signaling Technology | 4508 | 1:1000 |
| Anti-TAK1 | Cell Signaling Technology | 4505 | 1:1000 |
| Anti-Fibroblast | Santa Cruz | sc-46800 | 1:500 |
| Anti-GAPDH | Santa Cruz | sc-32233 | 1:1000 |
